# Alopecia areata following COVID-19 vaccine: a systematic review

**DOI:** 10.1186/s40001-024-01956-8

**Published:** 2024-07-05

**Authors:** Yunxia Zhu, Xiaoliang Ouyang, Deng Zhang, Xiuping Wang, Liang Wu, Simin Yu, Yanping Tan, Wei Li, Chunming Li

**Affiliations:** 1 Department of Dermatology, The Second Affiliated Hospital, Jiangxi Medical College, Nanchang University, 1 Minde Road, Nanchang, 330006 Jiangxi China; 2 Department of Plastic Surgery, The Second Affiliated Hospital, Jiangxi Medical College, Nanchang University, Nanchang, 330006 China; 3 Department of Dermatology, Jiangxi Provincial Maternal and Child Health Hospital, Nanchang, 330006 China; 4 Department of Dermatology, Second Affiliated Hospital, Zhejiang University School of Medicine, Hangzhou, 310000 China

**Keywords:** Alopecia areata, Adverse reactions, COVID-19 vaccines, SARS-CoV-2, Immunity

## Abstract

**Background:**

To date, multiple cases of adverse reactions to COVID-19 vaccines have been reported worldwide. Alopecia areata (AA) is an uncommon type of adverse reaction reported in some articles and has a significant social and psychological impact on patients. Our study aimed to review the AA and COVID-19 vaccine literature.

**Methods:**

This systematic review was conducted by searching for articles on AA following COVID-19 vaccines in international databases such as Embase, MEDLINE, PubMed, Web of Knowledge, and Ovid from December 2019 to December 30, 2023. We included studies that provided data for AA patients following COVID-19 vaccination with at least one dose. Data on sex, age, country/region of origin, vaccine type, days between vaccination and symptom presentation, manifestations of AA, trichoscopy and histopathological findings, treatment, and outcomes were included.

**Results:**

In total, 579 explored studies were identified and assessed, and 25 articles with a total of 51 patients were included in the review. Twenty-seven (52.9%) patients developed new-onset AA following receiving the COVID-19 vaccine, and AA recurrence or exacerbation occurred after receiving the COVID-19 vaccine in 24 (47.1%) patients with preexisting disease. Five vaccines were reported to cause AA in all cases. The Pfizer vaccine (45.1%) was the most frequently reported, followed by the ChAdOx1 nCoV-19 vaccine (27.5%), Moderna mRNA-1273 (19.6%), Sinopharm (3.9%) and SinoVac (3.9%). AA occurred most frequently within one month after the 1st dose, and then, the incidence decreased gradually with time. Topical or systemic corticosteroids were used in 38 patients. Eleven patients were treated with a Janus Kinase inhibitor (jakinib) inhibitor, eight with tofacitinib, and three with an unspecified jakinib. However, 3 of the 11 patients experienced exacerbations after treatment.

**Conclusion:**

AA after COVID-19 vaccination is rare, and physicians should be aware of this phenomenon to improve early diagnosis and appropriate treatment.

**Supplementary Information:**

The online version contains supplementary material available at 10.1186/s40001-024-01956-8.

## Introduction

With COVID-19 sweeping the globe, many measures have been applied for prevention and treatment. As one of the most effective measures, vaccines have been widely used in many countries. Over 180 vaccine candidates use a variety of technological platforms, including viral vectors, live attenuated virus, inactivated virus, virus-like particles, nucleic acid (DNA and RNA), peptides, and recombinant protein approaches, which have received approval for use or in development [1].

Although the abovementioned vaccines are generally safe, many articles about the side effects of these vaccines have been published. COVID-19 vaccines can induce various side effects, including headache, nausea, vomiting, fever, fatigue, itching, muscle pain, joint pain, local redness or swelling, and, rarely, anaphylactic shock [2]. Studies have also reported the development or recurrence of alopecia areata (AA) after COVID-19 vaccination. AA is an organ-specific autoimmune disorder characterized by nonscarring hair loss involving the scalp, face, or body. It can affect approximately 2% of the general population and cause severe psychological distress [2, 3]. Spontaneous hair regrowth occurs in approximately 80% of patients within one year after the onset of AA [4]. However, a small number of individuals can develop alopecia universalis (AU), which cannot be recovered in the short term and has a significant social and psychological impact on patients.

Three [5–7] systematic reviews, all published in 2022, explored the subject of COVID-19 vaccine-induced AA. The latest review included 13 studies. Since then, numerous new studies have been published in the literature on this subject. Hence, an updated review of the literature is warranted. The aim of our study was to provide a systematic review of this subject.

## Materials and methods 材料与方法

The present study was conducted based on the PRISMA (Preferred Reported Items for Systematic Review and Meta-analysis) guidelines. The review protocol was not registered with any groups such as Cochrane or Prospero. A completed copy of the PRISMA checklist (PRISMA 2020) has been added as an appendix file 1. The overall procedure can be divided into four-step selection process of identification, screening, eligibility, and inclusion. The literature screening, data extraction and quality assessment were done independently by two authors. Any disagreements were resolved by discussion or by a third author.

### Search strategy

A systematic search was conducted in the Embase, MEDLINE, PubMed, Web of Knowledge, and Ovid databases from December 2019 to December 30, 2023. No restrictions regarding study design, geographic region, or language were applied. A manual search of references cited in the selected articles and published reviews was also used for undetected studies. The search strategy is provided in detail Appendix file 2.

### Eligibility criteria

We included studies that provided data for case reports and case series of AA following COVID-19 vaccination with at least one dose. Review articles, non-peer reviewed sources and abstracts submitted in conferences were not eligible for inclusion. Studies on in vitro and animal models were excluded. Studies without mention of hair involvement after COVID-19 vaccination were excluded [8]. Studies assessing other types of nonscarring alopecia (i.e., telogen effluvium) and scarring alopecia were excluded.

### Data extraction and handling

Two independent reviewers screened titles and abstracts, followed by full-text articles. Discussions were used to settle disagreements. The following details of each article were recorded: sex, age, country/region origin, vaccine type, interval days between vaccination and symptom presentation, manifestations of AA, trichoscopy and histopathologic findings, treatment, and outcomes.

### Statistical analysis for evidence synthesis

Descriptive statistics were used to detail clinical characteristics of the patients. In case of normally distributed variables means, frequencies and mean ± standard deviation (SD) were used. For categorical data, percentages were displayed.

### Causality assessment of AEFI figure

An AEFI CA tool developed by WHO was applied to assess the causality assessment between COVID-19 vaccination and AA reported in the articles. All results were divided into four main categories includes (A) consistent with causal association to immunization; (B) indeterminate; (C) coincidental association; or (D) unclassifiable. Each AEFI report was evaluated separately by two clinicians with specific expertise in vaccinology, and differences in causality assessment’s outcomes were resolved via consensus.世卫组织开发的AEFI CA工具被用于评估文章中报告的COVID-19疫苗接种与AA之间的因果关系评估。所有结果分为四大类，包括：（A）与免疫接种的因果关系一致;（二）不确定的;（三）巧合关联;或 （D） 不可分类。每份AEFI报告均由两名具有疫苗学特定专业知识的临床医生分别评估，因果关系评估结果的差异通过协商一致解决。

## Results

### Study characteristics

**Fig. 1:**
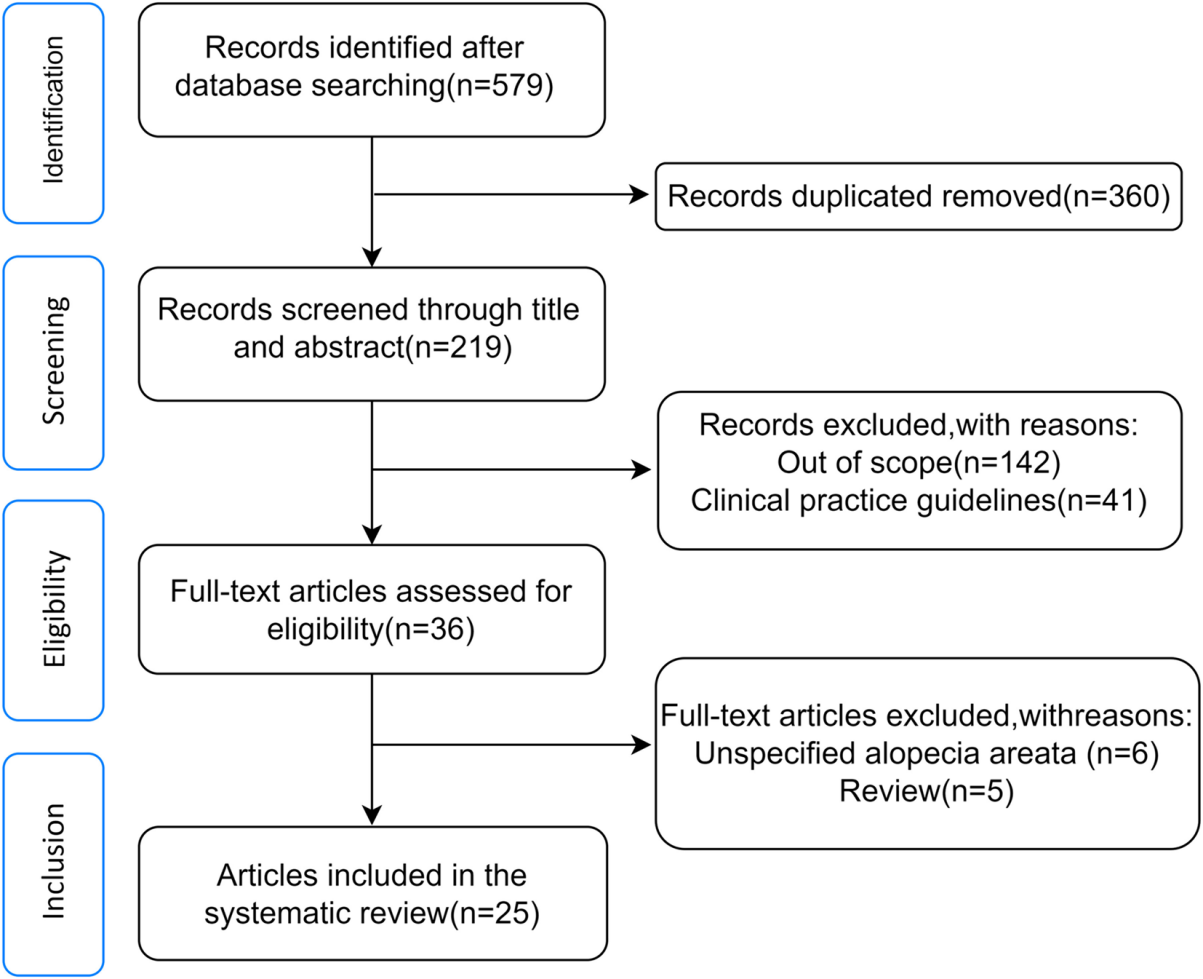
Literature search and article selection

The PRISMA flow diagram is available in Fig. 1. Through the initial searches, we identified a total of 579 potentially relevant articles. After removing 360 duplicates, 219 articles remained. By screening the titles and abstracts, 194 articles were excluded. The full texts of the remaining 25 articles were assessed for eligibility. The studies and clinical characteristics are summarized in Table 1 [3, 4, 6, 7, 9–29]. These studies originated from different countries or regions; eight case reports from Italy, Jamaica, Qatar, Japan, mainland China, Iran, America, Taiwan; four case series from America, Switzerland, California, and Italy; four letters to the editor from Egypt, Italy, and Colombia; three letters from America, Brazil and Taiwan; three correspondences from Italy, Taiwan and Japan; two articles from Iran and Italy; and one commentary from Taiwan.

**Table 1:** Clinical and demographic characteristics of the recruited patients

Author/ country or region	Age/ Sex	New or recurring cases	Past medical history and complication	Family history of AA or other autoimmune diseases	Vaccine	Interval days between vaccination and symptom presentation	Manifestations of alopecia areata	Trichoscopy and histopathologic findings	Treatment	Outcomes	Causality classification of AEFIs
Reham Essam/ Egypt	32/F	Recurring case	A previous mild attack of AA 6 years ago; a pervious COVID infection one year ago	NA	ChAdOx1	Few days after 1st dose	A hairless patch involving the scalp	Trichoscopy showed black dots, broken hairs, and newly growing hairs with some exclamation mark hairs	NA	NA	C
Giuseppe Gallo/ Italy	31/M	New case	None	None	Pfizer	Second day after 2nd dose	Multiple circular patches of alopecia on the occipital, bilateral parieto-temporal, and frontal areas, with involvement of the beard	Trichoscopy showed yellow-dots, black dots, dystrophic hair, and vellus hairs in the center and periphery of the patches	NA	NA	B
Margaret E. Scollan/ America	33/F	New case	Hepatic steatosis, chronic hepatitis B virus	Brother with AA	Moderna	2 months after 2nd dose	Large patches of nonscarring alopecia of the scalp with foci of hair regrowth	NA	ILTAC, pimecrolimus 1% cream, clobetasol 0.05% foam; Tofacitinib citrate 5 mg twice a day	Decreased hair loss and increased regrowth were noted	B
	57/F	Recurring case	Remote history of AA	NA	Pfizer	4 months after 2nd dose	Widespread nonscarring alopecia of the scalp with foci of hair regrowth	NA	Compounded tofacitinib 2%, clobetasol 0.05% ointment, clobetasol solution, Tofacitinib citrate 5 mg twice a day	Little improvement before tofacitinib therapy	C
	62/F	Recurring case	Remote history of AA	NA	Moderna	2 months after 2nd dose	*AU	NA	Tofacitinib citrate 10 mg twice a day, bimatoprost 0.03% eye drops	NA	C
	28/F	Recurring case	AA, Hashimoto thyroiditis	NA	Pfizer	Within 1 week after 2nd dose	*AU	NA	ILTAC and PRP; Tofacitinib citrate 10 mg twice a day	Little improvement before tofacitinib therapy	C
	29/F	New case	Elevated levels of TPOAb and TgAb antibody	NA	Pfizer	Within 1 week after 2nd dose	Two patches of nonscarring alopecia of the scalp with areas of regrowth	NA	ILTAC	NA	C
	22/M	New case	Elevated thyroid antibody	NA	Moderna	1 month after 2nd dose	Patches of nonscarring alopecia; 30% hair loss from scalp, 80% hair loss from beard	NA	ILTAC; Tofacitinib citrate 10 mg twice a day	Limited improvement before tofacitinib therapy	C
	15/M	New case	None	Grandmother with Hashimoto thyroiditis, sister with elevated thyroid antibod	Pfizer	Within 1 week after 2nd dose	Two patches of nonscarring alopecia of the scalp	NA	ILTAC	NA	B
	61/M	New case	Joint pain treated with hydroxychloroquine	NA	Pfizer	2 weeks after 1st dose	*AT	NA	Pending possible trial of oral tofacitinib citrate	NA	A
	16/M	New case	NA	NA	Pfizer	Within 1–2 weeks after 1st dose	*Patches of alopecia with 70% loss of scalp hair; sparse eyebrows and eyelashes	NA	ILTAC; Tofacitinib citrate 10 mg twice a day	Limited improvement before tofacitinib therapy	A
Marco May Lee/ Italy	80/M	New case	None	None	Pfizer	7 days after 1st dose	*After the first dose: beard hair loss on the left cheek and the upper lip with a concomitant widespread involvement of the entire scalp (SALT 65%); after the second dose: two months later observed AT	Ttrichoscopy showed cadaveric and exclamation point hairs were noted upon	Topical application of clobetasol foam; squaric acid dibutylester combined with topical 5% minoxidil	No improvement with topical application of clobetasol foam; 1 month from the start of immunotherapy no improvement was noted	A
Jonathan D. Ho/ Jamaica	51/F	New case	None	None	ChAdOx1	3 days after 1st dose	*Rapid loss of scalp hair, leading to near-complete baldness within 3 weeks, Axillary, pubic, and limb alopecia was also noted 12 weeks later (AU)	Histological examination showed peribulbar lymphocytic inflammation, approximately 30% of hairs in catagen/telogen phase, follicular structures failing to form hair. Dermatoscopy revealed broken hairs, yellow dots, and occasional exclamation mark hairs;	Clobetasol propionate ointment under occlusion and intralesional triamcinolone acetonide 10 mg/mL; oral tofacitinib	Had areas of sparse white regrowth and loss of exclamation mark hairs	A
Angela Lo/ America	61/F	New case	None	None	Pfizer	1 week after 2nd dose	4 areas of patchy hair loss localized to the scalp	Histological examination showed follicular miniaturization, a marked catagen/telogen shift, and a peribulbar, predominantlylymphocytic inflammatory infiltrate	Topical fluocinonide,topical minoxidil,topical tacrolimus 0.1% ointment, minoxidil 5% solution, Intralesional triamcinolone acetonide	Improvement was seen 1 month after treatment with full hair growth in all 4 areas at the 4-month follow-up	B
Federico Bardazzi/ Italy	41/F	Recurring case	Chronic recurrent patchy AA	NA	Pfizer	1 week after 1st dose	Patchy (multiple patches, SALT 26%)	Showed features of disease activity, including black dots, broken hairs, and exclamation mark hairs	Clobetasol proprionate foam 0.05% 5 times/week	Complete regrowth in 3 months	C
	24/M	Recurring case	Remote history of AA	Sister with androgenetic alopecia and AA incognita	Pfizer	1 week after 1st dose	*AT (SALT 100%)	The same as above	Intramuscolar triamcinolone; clobetasol proprionate 0.05%	Alopecia universalis. Scheduled for topical squaric acid dibutyl ester therapy	C
	21/F	New case	Allergic asthma	NA	Moderna	2 weeks after 1st dose	Patchy (multiple patches, SALT 42%)	The same as above	Injection of intralesional triamcinolone	Partial hair regrowth, reduction of activity signs at trichoscopy	A
Alfredo Rossi/ Italy	76/F	Recurring case	With personal history of AA	NA	Pfizer	2 weeks after 1st dose	Widespread hair loss was evident on the whole scalp, Eyebrows and eyelashes were preserved, and the hairs on other body sites were normal	Trichoscopy showed many black dots and broken hairs	Topical treatment with steroids	NA	C
	59/F	Recurring case	With autoimmune thyroiditis and two previous episodes of patchy AA; referred mild fever and arthralgia the day after 1st dose	NA	ChAdOx1	3 weeks after 1st dose	An oval bald patch localized at the vertex	Trichoscopy showed an oval bald patch localized at the vertexTrichoscopy showed numerous black dots and broken hairs	Topical steroids and oral vita-min D	NA	C
	29/F	Recurring case	Had an episode of patchy AA	NA	ChAdOx1	2 weeks after 1st dose	Had sudden and progressive hair loss, generalized hair loss was evident on the scalp with partial loss of eyebrows and eyelashes	Trichoscopy showed multiple black dots and broken hairs	Topical treatment with steroids	NA	C
Hsuan-An Su/ Taiwan	42/M	New case	None	None	ChAdOx1	3 weeks after 1st dose	Several well-demarcated patches of hair loss on the scalp without scarring or scaling measuring from 1 to 10 cm in diameter	Dermoscopy demonstrated yellow dots, black dots, short vellus hairs, exclamation mark hairs, and tapering hair	Intralesional injections of 10 mg/dL triamcinolone	NA	A
Safoura Shakoei/ Iran	74/M	New case	Fatty liver	NA	Sinopharm	2 days after 2st dose	Scalp and beard area alopecia	NA	Intralesional Corticosteroid injection	Significant improvement	B
	37/M	New case	None	NA	Sinopharm	6 days after 1st dose	Beard area after the first dose that progressed into scalp and eyebrow alopecia (mild symptoms after the first does that worsened after the second dose)	NA	Intralesional Corticosteroid injection	Significant improvement	A
Shirley Braga Lima Gamonal/Brazil	27/F	New case	Diagnosed with SLE 3 weeks after receiving 2st dose of ChAdOX1	None	Pfizer	15 days after 3rd dose	Diffuse alopecia (involving the temporo-parietal, occipital, and vertex areas)	Trichoscopy showed yellow dots, black dots, dystrophic hair, and white hairs of repilation. Histological examination showed a mild lymphocytic infiltrate around the outer follicular sheath without signs of fibrosis	NA	NA	B
Chieh-Hsun Chen/Taiwan	29/M	Recurring case	Had a history of AA, the disease has been stable for 7 months without active hair loss (SALT 13%)	NA	ChAdOx1	1 week after 2st dose	*Diffuse hair shedding across the scalp (SALT 82%)	NA	Pulse steroid therapy	The disease continued to progress during 2-month follow-up	C
	26/F	Recurring case	Previously diagnosed with AA and nearly complete remission preceding the vaccination for 2 months (SALT 5%)	NA	Pfizer	2 weeks after 2st dose	*Rapid progression to AU (diffuse hair loss involving the scalp, eyebrows, eyelashes, and body hairs)	NA	Pulse steroid therapy	No response despite four courses of treatment	C
Hani Abdalla/QAT	63/F	Recurring case	Remote history of AA, presented with hypothyroidism, prediabetes and thalassemia trait when came to the hospital	None	Pfizer	Within 1 week after 1st dose	*Patchy hair loss within two weeks from the first dose, the second dose resulted in AU	NA	NA	NA	C
Fabrizio Martora/ Italy	7/F	New case	Complicated with herpes zoster	NA	Pfizer	20 days after 2nd dose	A nonscarring alopecic patch localized on occipital region with foci of hair regrowth	NA	NA	NA	B
Yoshihiro Matsuda/Japan	37/F	New case	Childhood atopic dermatitis;persistent low-grade fever (37.2 °C) on day + 13 after the first dose	NA	Pfizer	22 days after 1st dose	*Noticed coin-sized hair loss on day 22 after the first dose;widespread alopecia (SALT 80%) on day73 after the first dose	Trichoscopy showed tapering hairs, broken hairs, black dots, and increase in vacant follicular ostia; Histological examination revealed perifollicular lymphocytic infiltrate and increased number of telogen hairs	Topical betamethasone butyrate propionate lotion	Mostly recovered leaving only one oval bald patch on day + 310 after the first dose (SALT 5%)	A
Kentaro Iwata/Japan	40/F	New case	A mild fever for a few days after received the second dose	None	Moderna	1 week after 1st	*AU	NA	Oral prednisone of an unknown dosage; excimer lamp treatment; oral cepharanthin, and monoammonium glycyrrhizinate; Shimotsuto	Alopecia improved a little	C
Jenny Hernández Arroyo/ Switzerland	27/F	Recurring case	Had a history of AU and polycystic ovary 6 years ago	None	Pfizer, SinoVac, and ChAdOx1 (Booster)	8 days after 3rd (Booster)	*AU	NA	Mesotherapy and pulses of dexamethasone and clobetasol propionate 0.5% topical	25% of improvement	C
	51/F	New case	Arterial hypertension	Grandmother with hypothyroidism	Pfizer, SinoVac, and ChAdOx1 (Booster)	3 days after 3rd (Booster)	*AU	NA	Mesotherapy	80%of improvement	B
	34/M	New case	COVID-19	None	Pfizer, SinoVac, and ChAdOx1 (Booster)	10 days after 3rd (Booster)	Telogen effluvium	NA	Mesotherapy	50%of improvement	B
	40/M	New case	COVID-19	None	Pfizer, SinoVac, and ChAdOx1 (Booster)	7 days after 3rd (Booster)	Telogen effluvium	NA	Mesotherapy	90%of improvement	B
	59/M	New case	Arterial hypertension; COVID-19	None	Pfizer, SinoVac, and ChAdOx1 (Booster)	17 days after 3rd (Booster)	*AU	NA	Mesotherapy and pulses of dexamethasone and clobetasol propionate 0.5% topical	15%of improvement	B
Yi WU/ mainland China	20/F	New case	None	None	SinoVac	2 weeks after 3rd (Booster)	Multiple hairless patches throughout the scalp (SALT 30%)	Dermoscopy examination revealed broken hairs, black dots, and some exclamationmark hairs	Combination of topical and oral steroids	NA	B
Arish Babadjouni/California	33/F	Recurring case	Moderate-to-severe alopecia areata (SALT 66%) before vaccination	NA	Pfizer	within 2 weeks after 1st dose	*AT (SALT 99%)	NA	Systemic JAKi therapy	The hair loss persisted with minimal changes (SALT 99%) at 3 months of treatment, total SALT increased 33% during the therapeutic process	C
	27/M	Recurring case	Moderate-to-severe alopecia areata before (SALT 33%) 2nd dose vaccination	NA	Pfizer	2 weeks after 2nd dose	* (SALT 59%)	NA	Systemic JAKi therapy	Total SALT increased 25% during the therapeutic process	C
	32/M	Recurring case	Moderate-to-severe alopecia areata before (SALT 62%) vaccination	NA	Moderna	2 weeks after 1st dose	* (SALT 70%)	NA	Systemic JAKi therapy	Total SALT increased 8% during the therapeutic process	C
Zakiye Ganjei/Iran	23/F	New case	Myalgia following the vaccination	None	ChAdOx1	1 week after 1st dose	*AT	Histological examination revealed peribulbar lymphocyte infiltration with increased miniaturized hairs	Betamethasone cream 0.1% and pimecrolimus cream 1%; Systemic corticosteroid (oral prednisolone, 300 mg monthly, for 3 months	NA	A
	26/F	Recurring case	With a history of AA; infection with COVID-19 one year ago	NA	ChAdOx1	2 weeks after 2nd dose	*AT	NA	Systemic corticosteroid was initiated (oral prednisolone, 300 mg monthly, for 3 months)	NA	C
Lucia Genco/Italy	25/F	Recurring case	With a history of AA 4 years ago	NA	Moderna	1 week after 1st dose	*SALT (S1) after first dose; SALT (S2) after second dose; SALT after (S3) third dose	Trichoscopic examination of the newest patches showed black dots; broken hair; yellow dots	Minoxidil 5% 1 mL bis in die, topical clobetasol, topical growth factors, and ILTAC 3:1	Did not benefited from the therapy until the statistics are completed	C
	23/F	Recurring case	Had mild alopecia at baseline	NA	Moderna	2 weeks after 1st dose	SALT (S1) before vaccination; SALT (S2) after first dose; SALT (S2) after second dose; SALT after (S2) third dose	The same as aboves	The same as above	The same as above	C
	32/F	Recurring case	With a history of AA 2 years ago	NA	Pfizer	2 weeks after 1st dose	*SALT (S3) after first dose; SALT (S3) after second dose	The same as above	The same as above	The same as above	C
	31/M	Recurring case	With a history of AA 2 years ago	NA	Pfizer	3 weeks after 1st dose	SALT (S1) after first dose; SALT (S1) after second dose; SALT after (S1) third dose	The same as above	The same as above	The same as above	C
	51/F	Recurring case	With a history of AA 1 year ago	NA	Moderna	2 weeks after 1st dose	SALT (S1) after first dose; SALT (S1) after second dose	The same as above	The same as above	The same as above	C
Fatmah AlZahrani/America	44/M	New case	COVID-19 Infection 3 months ago	None	Moderna	2 weeks after 1st dose	Developed patchy areas of hair loss 2 weeks after first dose; experienced diffuse hair loss on his scalp, eyelashes, beard hair, and eyebrows 1 week after second dose	NA	Prednisone 50 mg PO daily, betamethasone valerate 0.05% lotion	Marie Antoinette syndrome	A
Marta Fusano/ Italy	42/M	Recurring case	Had a previous single episode of patches AA 2 years ago; atopic dermatitis and celiac disease	NA	Pfizer	After 2nd dose	*Experience a patches alopecia after second dose; AT after third dose	Trichoscopy shows prevalence of yellow dots	Topical treatment with high potency steroids	NA	C
	18/M	Recurring case	AA	None	Pfizer	20 days after 1st dose	*Patches of AA before vaccination; experienced a mild worsening of the patches after first dose; AT after second dose	Trichoscopyshows yellow dots, black dots, vellus hairs and exclamation mark hairs	Topical treatment with high potency steroids	NA	C
Hsiang-Chiun Teng/Taiwan	34/F	New case	None	NA	ChAdOx1	4 weeks after 2nd dose	Hair loss over the entire scalp	NA	Oral prednisolone, 25–40 mg daily for more than a month; PRP therapy	No response to the steroid treatment; The hair loss stopped and new hair regrew after two doses of PRP treatment, and recovered completely after six courses of PRP treatment	B
Miguel Aristizabal/Colombia	33/F	New case	None	None	Sinovac	One month after 2nd dose	The scalp showed several delineated hair loss patches with no scarring or scaling over the vertex, bitemporal,and occipital region, compromising < 50% of the scalp	Trichoscopy showing broken and exclamation mark hairs, black dots, yellow dots, and newly growing hairs	Intralesional and topical corticosteroids	Lesions did not progress and signs of regrowth were noted at 1-month clinical follow-up	B

M = male, F = female; NA = not applicable; AA = Alopecia areata; AU = Alopecia universalis; AT = Alopecia totalis; ILTAC = intralesional triamcinolone; PRP = platelet-rich plasma; SALT = Severity of Alopecia Tool; A = classification includes consistent with causal association to immunization; B = indeterminate; C = coincidental association; D = unclassifiable. Severe involvement of alopecia (scalp hair loss ≥ 50%) is marked by *

### Patient characteristics

In total, 51 patients, including 31 females (60.7%) and 20 (39.3%) males, were included. The average age of these patients was 37.6 years (37.6 ± 16.5 years). Seven patients (13.7%) were aged older than 60 years, four (7.8%) were aged younger than 20 years, and the remaining forty (78.5%) were aged between 20 and 60 years. 27 patients (52.9%) developed new-onset AA following COVID-19 vaccination, while 24 patients (47.1%) presented with relapsed or aggravated AA. Ten patients (19.6%) complicated with other atopy or autoimmune diseases, including six patients (11.8%) with thyroiditis or elevated thyroid antibody levels, two with atopic dermatitis (AD), one with systemic lupus erythematosus (SLE), and one with asthma. Six patients (11.8%) had a history of COVID-19 infection. Furthermore, two patients (3.9%) had a family history of AA, and two patients (3.9%) had a family history of thyroid dysfunction.

### Characteristics of reaction

In our study, Pfizer was the most common AA vaccine, and 23 (45.1%) of the 51 cases were caused by Pfizer, followed by ChAdOx1, nCoV-19 (27.5%), Moderna mRNA-1273 (19.6%), Sinopharm (3.9%) and SinoVac (3.9%).

Twenty-six of the 51 (50.9%) patients experienced hair loss within one month after the 1st dose, 13 of the 51 (25.5%) experienced hair loss within one month after the 2nd dose, seven of the 51 (13.7%) experienced hair loss within one month after the 3rd dose, three of the 51 (5.9%) experienced hair loss within two to three months after the 2nd dose, one of the 51 (2%) experienced hair loss within one to two months after the 2nd dose, and one of the 51 (2%) experienced hair loss within three months after the 2nd dose. In general, the incidence decreased gradually over time. Among the 51 patients, 34 patients (66.7%) experienced a patchy AA, while eight patients (15.6%) progressed to alopecia totalis (AT), and nine patients (17.7%) progressed to AU.

### Treatment and outcome

Among the 46 patients who received documented treatment, 38 received topical or systemic corticosteroids. Intralesional triamcinolone acetonide was used in 16 patients [4, 12–15, 24], methylprednisolone pulse steroid therapy in two patients [30], pulses of dexamethasone in two patients [21], and oral prednisolone 25–300 mg daily in five patients [7, 20, 25]; the other patients were treated with various potent topical corticosteroids. In addition, seven patients were treated with 5% minoxidil solution [11, 13, 24], five patients with mesotherapy [21], five with topical growth factors [24], one with squaric acid dibutyleste [11], two with pimecrolimus 1% cream [4, 7], and one with 0.1% tacrolimus topical ointment [13]. Two patients [4, 27] received platelet-rich plasma therapy (PRP). Notably, 11 patients were treated with a jakinib, eight patients [4, 12] were treated with tofacitinib, and three patients were treated [23] with unspecified jakinib for severe AA or refractory (corticosteroid was ineffective) AA [4, 27].

Among the patients who mentioned the outcome of treatment, ten patients [10, 13, 14, 16, 19, 21, 27] had good treatment effects, with reduced hair loss, increased regeneration, or complete regeneration. Interestingly, one patient [25] experienced rapid diffuse hair loss with subsequent depigmentation 2 weeks after the first vaccination but spontaneous total regrowth with snow-white hair in the end. However, the therapeutic efficacy was poor in ten patients. One patient [14] did not respond to hormone therapy and progressed from AT to UT during treatment. Two [30] patients failed to respond to pulsed steroid therapy. One patient [11] showed no improvement after one month of immunotherapy. Many therapies, such as oral prednisone, an excimer lamp, oral cephalothin, and mono ammonium, were used in one [20] patient but still led to little improvement. One [4] patient had little improvement after the treatment of compounded tofacitinib 2%, clobetasol 0.05% ointment, and clobetasol solution. One [4] received ILTAC and platelet-rich plasma therapy, but still leaded to AU. In addition, three patients [23] with moderate-to-severe AA experienced various degrees of exacerbation of hair loss despite therapy with jakinib, one with an increase in total severity of alopecia tool (SALT) of 33%, one with an increase of 25%, and one with an increase of 8%.

### Causality assessment

According to the causality assessment, 10/51 (19.6%) were classified as “consistent with causal association to immunization” to the COVID-19 vaccination, 14/51 (27.4%) were “indeterminate”, and 27/51 (53%) were classified as “coincidental association”; no case was considered as “unclassifiable” (Table 1).

## Discussion

The present study investigated the current literature on cases of AA following COVID-19 vaccination. Barahmani et al. [31] revealed that women exhibit a higher rate (72%) of AA in a large case–control study. Similarly, our study discovered that women have a comparatively higher prevalence rate (60.7%) of AA. This may be due to women’s higher susceptibility to autoimmune diseases. Moreover, AA typically occurs at a younger age (21–40 years) [32]. Hence, the incidence of AA in elderly individuals is relatively rare. Jang et al. [33] reported that 3.5% of the 1,761 newly diagnosed patients with AA were over 60 years old. However, in our study, 13.7% of the described patients were older than 60 years. Therefore, the relationship between age and the incidence of AA in elderly individuals needs further investigation.

Of note, our study showed that 24 patients (47.1%) had a history of AA, while six patients (11.8%) had thyroid disease. The incidence of thyroid disease has varied from 8 to 28% in patients with AA [34]. Noso et al. [35] have previously reported an association between thyroid autoimmunity and AA. In their study, they found a positive association of DRB1*15:01-DQB1*06:02 with AA in thyrotropin (TSH) receptor antibody-positive patients, indicating a common etiology and susceptibility between AA and autoimmune thyroiditis. Hence, our study highlights that patients with a history of AA or thyroid dysfunction may have a greater risk of AA following COVID-19 vaccination.

In our study, the most common of the reported vaccines was the Pfizer vaccine, an mRNA vaccine. We deduce that this may be due to the following reasons. Firstly, previous studies have shown that the majority of cutaneous adverse reactions are reported after mRNA-based vaccines. It is believed that polyethylene glycol (PEG) may be one of the causes of allergic reactions in mRNA vaccines [36]. Secondly, it is also possible that Pfizer's vaccine was approved earlier and adopted more widely around the world. In addition, according to our findings, AA occurs most frequently within one month after the 1st dose and then shows a gradual downwards trend over time. This observation is consistent with the report by Qaderi et al. [37] Hence, increased monitoring for AA may be warranted during the first 4 weeks following COVID-19 vaccination.

To date, different treatment options (local and systemic) are available, but none of them can guarantee that patients with AA can fully recover or without relapse [19, 20]. A few patients with severe AT have a poor prognosis, and the condition can persist for a long time. Potent topical glucocorticosteroids or systemic immunosuppressants need to be sustained for more than three months to have a significant therapeutic effect [21]. JAK pathway plays a key role in the pathophysiology of AA and is a potential target for treatment. Even in patients who fail conventional treatment, oral jakinib therapy can promote significant hair regrowth. However, three patients experienced varying degrees of exacerbation after treatment with jakinib. Hence, whether other jakinibs with different selectivity for JAK subtypes may still be beneficial in these patients deserves further investigation in the future.

In eight [4, 6, 11, 16, 24–26] of the 51 patients, AA occurred after the first vaccination and became significantly worse after the next dose. Four specific cases worth mentioning are presented below. Three patients [11, 26] developed patchy areas of hair loss after the first vaccination but were still inoculated with the next dose, which eventually resulted in AT. In addition, Abdalla [6] et al. reported the case of a 63-year-old woman who developed patchy hair loss after the first dose of the vaccine. The second dose resulted in increased hair loss, followed by complete hair loss of the entire body hair. We speculate that the first dose may have triggered the autoimmune response, while the second, as performed on an already sensitized immune system, may have boosted the autoimmune attack on the hair bulb, leading to a marked worsening of the condition. This indicates that dermatologists should consider carefully whether to receive a second dose if a patient develops symptoms of AA after the first dose of COVID-19.

The exact pathogenesis by which COVID-19 vaccination induces AA is unclear. Vaccine-associated autoimmunity due to either cross-reactivity between antigens or the effect of adjuvants is a well-known phenomenon. mRNA vaccines such as Pfizer BNT162b2 can lead to the self-production of antigenic proteins that can stimulate the host immune response. As a result, proinflammatory cascades are activated, and numerous cytokines, including interferon (IFN) and interleukin (IL)-6, are released. IL-6 can inhibit the proliferation of hair follicle stem cells and keratinocytes and the transition of hair follicles from telogen to anagen [3, 38]. IFN can cause the collapse of immune privilege in human follicles [39, 40]. In addition, based on an adenoviral vector delivering the gene encoding the spike protein, the AZD1222/ChAdOx1 vaccine shares the same goal of evoking T cell-mediated immune reactions [41]. However, further studies elucidating the genesis of vaccine-associated AA are needed.

### Limitations

This study has several limitations. First, a small number of cases had missing data for treatment and outcomes. Furthermore, the current study was subject to possible diagnostic bias. When a particular adverse event such as AA is suggested and publicized to be associated with a vaccine, it can result in preferential identification of cases due to increased awareness. Lastly, these studies mainly consisted of case reports and case series with limited research data. Therefore, future studies with robust designs, such as cohorts, are needed to more accurately confirm these findings.

## Conclusions

Our study highlights that patients with a history of AA or thyroid dysfunction may be at higher risk of developing AA after receiving the COVID-19 vaccine. Furthermore, it was found that AA most commonly occurred within one month after the first dose and then gradually decreased over time. We hope to provide a reference for observing the timeframe within which AA may occur following COVID-19 vaccination. Moreover, some patients in our study experienced alopecia after the first dose but proceeded with the second dose, leading to more severe alopecia. Therefore, we recommend that dermatologists carefully assess whether to administer the second dose if alopecia symptoms arise after the initial COVID-19 vaccine dose, to prevent irreversible AA.

### Supplementary Information

Additional file 1.

Additional file 2.

## Data Availability

The data that support the findings of this study are available from the corresponding author upon reasonable request.
