# Supplementary material for: Alopecia areata following COVID-19 vaccine: a systematic review
Source: Eur J Med Res. 2024 Jul 5;29:356. doi: 10.1186/s40001-024-01956-8 (PMC11225413; doi:10.1186/s40001-024-01956-8)
Supplement: Supplementary file 2 — Additional file 2. [file 40001_2024_1956_MOESM2_ESM.docx]

**Supplementary Appendix file2: Search strategy (last search: 30t December 2023)**

| **Database** | **Search strategy** |
| --- | --- |
| PubMed | ((((((("SARS-CoV-2"[Mesh]) OR "COVID-19"[Mesh]) OR (COVID-19[Title/Abstract])) OR (Coronavirus[Title/Abstract])) OR (nCoV[Title/Abstract])) OR (SARS-Cov-2[Title/Abstract])) AND ((("Vaccines"[Mesh]) OR (vaccines[Title/Abstract])) OR (vaccin*[Title/Abstract]))) AND (((((Alopecia areata[Title/Abstract]) OR (Alopecia[Title/Abstract]))OR (Alopecia universalis[Title/Abstract])) OR (Alopecia totalis[Title/Abstract]))OR (Hair loss[Title/Abstract])) |
| Web of Science | ((TS=(SARS-CoV-2 OR SARS-CoV-2 Virus OR SARS-CoV-2 Viruses OR 2019 Novel Coronavirus OR 2019 Novel Coronaviruses OR Novel Coronavirus, 2019 OR COVID-19 Virus OR COVID 19 Virus OR COVID-19 Viruses OR Virus, COVID-19 OR Wuhan Coronavirus OR Coronavirus, Wuhan OR COVID19 Virus OR COVID19 Viruses OR Virus, COVID19 OR Viruses, COVID19 OR Coronavirus Disease 2019 Virus OR SARS Coronavirus 2 OR Coronavirus 2, SARS OR 2019-nCoV)) AND TS=((Alopecia areata OR Hair loss OR Hypotrichosis OR(Alopecia OR Alopecia universalis OR Alopecia totalis) )) AND TS=((Vaccine OR Vaccines OR Vaccination OR Vaccin*))) |
| EMBASE | ('coronavirus disease 2019':ab,ti OR 'covid 19':ab,ti OR 'coronavirus':ab,ti OR ncov:ab,ti OR 'sars cov 2':ab,ti) AND ('vaccine ':ab,ti OR 'vaccines':ab,ti OR 'vaccin* ':ab,ti) AND ('Alopecia areata ':ab,ti OR 'Alopecia universalis':ab,ti OR 'Alopecia totalis':ab,ti OR 'Hair loss':ab,ti)  ('covid 19'/exp OR 'covid 19' OR 'coronavirus disease 2019'/exp OR 'coronavirus disease 2019' OR 'coronavirus'/exp OR 'coronavirus' OR 'ncov' OR 'sars cov 2'/exp OR 'sars cov 2') AND ('vaccine'/exp OR 'vaccines'/exp OR 'vaccin*' OR 'vaccination'/exp OR 'vaccination') AND ('alopecia areata'/exp OR 'alopecia areata' OR 'alopecia universalis'/exp OR 'alopecia universalis' OR 'alopecia totalis'/exp OR 'alopecia totalis' OR 'hair loss'/exp OR 'hair loss') |
| MEDLINE | ((TS=(SARS-CoV-2 OR SARS-CoV-2 Virus OR SARS-CoV-2 Viruses OR 2019 Novel Coronavirus OR 2019 Novel Coronaviruses OR Novel Coronavirus, 2019 OR COVID-19 Virus OR COVID 19 Virus OR COVID-19 Viruses OR Virus, COVID-19 OR Wuhan Coronavirus OR Coronavirus, Wuhan OR COVID19 Virus OR COVID19 Viruses OR Virus, COVID19 OR Viruses, COVID19 OR Coronavirus Disease 2019 Virus OR SARS Coronavirus 2 OR Coronavirus 2, SARS OR 2019-nCoV)) AND TS=(Alopecia areata OR Hair loss OR Hypotrichosis OR(Alopecia OR Alopecia universalis OR Alopecia totalis)) AND TS=(Vaccine OR Vaccines OR Vaccination OR Vaccin*)) |
| Ovid | (SARS-CoV-2.sh. or covid 19.af. or coronavirus.af.or coronavirus disease 2019.af.or ncov.af.) and (vaccine.sh.or vaccines.af. or vaccin*.af.or vaccination.af.) and (alopecia areata.sh. or alopecia.af. or alopecia universalis.af.or alopecia totalis.af.or hair loss.af.) |
